# Adiponectin, leptin and insulin levels at birth and in early postnatal life in neonates with hypoxic ischemic encephalopathy

**DOI:** 10.1186/s40200-015-0219-1

**Published:** 2015-12-01

**Authors:** Abdel-Azeem M. El-Mazary, Khalid A. Nasif, Gehan L. Abdel-Hakeem, Tahra Sherif, Ebtesam Farouk, Ebtesam M. El-Gezawy

**Affiliations:** Pediatric Department, Faculty of Medicine, Minia University, Minya, Egypt; Biochemistry Department, Faculty of Medicine, Minia University, Minya, Egypt; Clinical pathology Department, Faculty of Medicine, Assuit University, Assuit, Egypt

**Keywords:** Adiponectin, Leptin, Insulin, SGA, AGA, Neonates, Hypoxic, Ischemic encephalopathy

## Abstract

**Background:**

Hypoxic ischemic encephalopathy (HIE) occurs in one to three per 1000 live full-term births. Fifteen to twenty percent will die in the postnatal period, and an additional 25 % will develop severe and permanent neuropsychological sequalae. The control of growth and nutritional status in the fetus and neonate is a complex mechanism, in which also hormones produced by adipose tissue, such as adiponectin and leptin are involved. The aim of this study was to measure the levels of adiponectin, leptin and insulin in neonates with HIE at birth and in early postnatal life and comparing them with normal healthy AGA and SGA neonates

**Methods:**

This study carried out on 80 full-term neonates born in Minia university hospital during the period from May 2013 to December 2014. They were divided into group I included 25 neonates with HIE and group II included 55 normal healthy neonates (30 appropriate for gestational age (AGA) and 25 small for gestational age (SGA)). Weight, length, head circumference, body mass index (BMI), glucose, adiponectin, leptin and insulin levels were measured for all neonates. Adiponectin, leptin and insulin levels were compared between neonates with HIE and normal healthy neonates as well as between AGA and SGA neonates at birth, 2nd and 6th days of life.

**Results:**

Adiponectin and leptin levels were significantly higher at birth then began to decrease during the first postnatal week in all neonates while insulin level increased during the same period. Serum adiponectin levels were significantly lower while serum leptin and insulin levels were significantly higher in neonates with HIE than healthy neonates. In all neonates, the serum adiponectin level was positively correlated at birth with weight, length, BMI and leptin levels but not with insulin level. In neonates with HIE, serum adiponectin level was not correlated with weight, BMI, leptin level or insulin level. In all neonates, the serum leptin level was positively correlated at birth with body weight, height and BMI. In neonates with HIE serum leptin levels were not correlated with weight, BMI or insulin level after birth. There were no correlations between either leptin or adiponectin serum levels or any of the studied parameters in neonates with HIE

**Conclusions:**

Neonates who are suffering from HIE had lower serum levels of adiponectin and higher serum levels of leptin and insulin than normal healthy neonates at birth and during the early postnatal period. The decline of leptin and increased the insulin levels after birth in all neonates may be important for the stimulation of feeding behavior and the acquisition of energy homeostasis during the early postnatal life. Positive significant correlations between adiponectin, leptin, body weight and body mass indices were present in normal healthy neonates but not in neonates with HIE reflecting the effect of hypoxia on the regulatory mechanisms controlling the adipose tissue functions.

## Background

Hypoxic ischemic encephalopathy (HIE) occurs in one to three per 1000 live full-term births [1]. About 15–20 % will die in the postnatal period, and an additional 25 % will develop severe and permanent neuropsychological sequalae [2]. According to the latest estimates by world health organization (WHO), 98 % of neonatal deaths take place in the developing countries. Perinatal asphyxia and birth injuries together contribute to almost 29 % of these deaths [3]. Hormones produced by adipose tissue, such as adiponectin and leptin play a critical role in the control of energy balance in both of childhood and adult life [4]. Recent data show that children may follow different growth patterns and that those who display excessively rapid body mass index (BMI) gains may be at higher risk for adult obesity and adverse metabolic health [4, 5]. Adiponectin, a protein hormone secreted by adipose tissue, targets muscle and liver to increase uptake and catabolism of fatty acids and carbohydrates, promoting insulin sensitivity [6]. Several studies reported a positive relationship between adiponectin and birth weight [7, 8] but others did not [9–11].

**Fig. 1:**
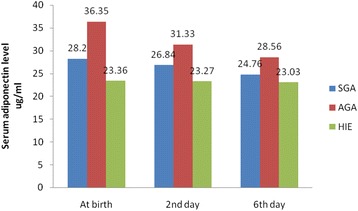
Circulating levels of adiponectin, in SGA, AGA and neonates with HIE at birth, 2nd, 6th days

**Fig. 2:**
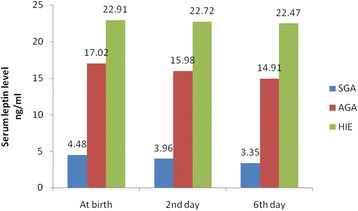
Circulating levels of leptin in SGA, AGA and neonates with HIE at birth, 2nd, 6th days

**Fig. 3:**
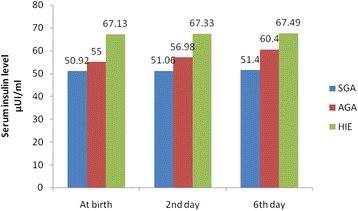
Circulating levels of Insulin in SGA, AGA and neonates with HIE at birth, 2nd, 6th days

Leptin, a hormone synthesized mainly by adipose tissue acts on the hypothalamus to convey satiety and regulate long-term energy balance [12]. There are many studies about the leptin role in energy and nutrition balance in human and mice [13–15]. It was hypothesized that children have a relative leptin resistance beneficial for their positive energy needs that occurs after birth [16].

Although, there were many studies about the effect of HIE on different tissue functions but its effect on the adipose tissue functions is not clear until now [17, 18].

The aim of this study was to measure the levels of adiponectin, leptin and insulin in neonates with HIE at birth and in early postnatal life and comparing them with normal healthy AGA and SGA neonates.

## Methods

### Study subjects and samples collection

A total of 80 full-term neonates (37–42 weeks) born in Minia university hospital during the period from May 2013 to December 2014 were enrolled in the study. They were divided into two groups: Group I included 25 neonates suffering from HIE and group II included 55 neonates apparently normal healthy neonates not suffering from any complications and matched with age and sex as controls (30 AGA and 25 SGA neonates).

Inclusion criteria for neonates with HIE [19, 20]: asphyxia was defined as the presence of three or more of the following: Profound metabolic acidosis (pH less than 7.00 and base deficit ≤ −12,mmol/L) on an umbilical cord arterial blood sample, early onset of severe or moderate neonatal encephalopathy in neonates born at 37 or more weeks of gestation with neurological dysfunction including altered level of consciousness and reactivity, altered tone, hyperactive or absent reflexes, weak or absent Moro reflex and seizures, sentinel hypoxic event during labor (heart rate of less than 100 beats per minute, late decelerations, or reduced beat-to-beat variability or, severe electronic fetal monitoring abnormalities, thick meconium stained amniotic fluid and respiratory depression, hypotonia, or bradycardia, a need for resuscitation for more than 3 min with positive pressure ventilation and oxygen immediately after birth, Apgar score of 0 to 3 beyond 5 min, onset of multisystem involvement within 72 h of birth and ∕or early imaging study showing evidence of acute non focal cerebral abnormality.

SGA was defined as body weight below 2.5 Kg. and gestational age at delivery was calculated according to last menstrual period and confirmed by ultrasound examination during the first or early second trimester.

Informed consents were obtained from all parents of neonates before inclusion in the study, for which local departmental research committee approval was obtained.

All groups were subjected to anthropometric measurements (weight, length and head circumference). Body mass index (BMI) was calculated from the formula: BMI = body weight (in Kg) / Length (in m^2^)

Blood samples [3 ml for each sample] were obtained from all neonates within the first 24 h, 2nd and 6th days of life. The obtained samples were centrifuged, and serum was separated immediately by centrifugation and stored at −20 °C until assays. Neonates with evidence of malformations, genetic disorders, early onset sepsis, twins and neonates of diabetic, hypertensive or pre-eclamptic mothers were excluded from the study. Serum glucose was determined using auto-analyzer BM/Hitachi. Serum adiponectin concentration was assayed with an adiponectin ELISA kit (Biovendor Laboratory Medicine, Inc., Czech Republic). Serum Leptin was assayed with Human Leptin ELISA kit (DRG international, Inc., Germany). Serum Insulin was assayed with human insulin ELISA kit (BioSource Europe S.A., Belgium). For the parameters measured, the intra-assay variation was 2.7–8.6 %, 2.8–7.2 % and 2.5–6.9 % and inter-assay variation of 12–28 %, 13.8–25.6 % and 11.6–15.5 % for adiponectin, leptin and insulin respectively. Minimum detectable concentrations (MDC) were as follows: adiponectin, 12.5 (μg/ml); leptin, 8.7 ng/ml and insulin, 22.4 (μIU/ml). [21–23].

### Statistical analysis

The results are presented as means ± SD. All calculations were made using the SPSS program (version 18). Spearman correlation coefficient was used instead of Pearson r correlation coefficient to compare individual changes over time. We used ANOVA test for statistical comparisons of baseline characteristics. Probability values of less than 0.05 were considered significant.

## Results

Serum adiponectin concentration was significantly lower while the leptin and insulin concentrations were significantly higher in neonates with HIE than normal healthy neonates. As expected, all neonatal anthropometric measurements were higher in the AGA than SGA group. (Tables 1 & 2) and (Figures 1-3).

**Table 1:** Anthropometric and laboratory data of studied groups at birth

Item	HIE	SGA	AGA	p-value
	(No.25)	(No. = 25)	(No. = 30)	
Gest. Age(w)	38.7 ± 1.6	38.8 ± 1.6	39.16 ± 1.8	NS
M/F ratio	18(72 %)/7(28 %)	19(76 %)/6(24 %)	22(74 %)/8(26 %)	NS
NVD/CS	11(44 %)/14(46 %)	13(52 %)/12(48 %)	16(54 %)/14(46 %)	NS
Weight (Kg)	3.11 ± 0.32	1.91 ± 0.32	3.44 ± 0.24	0.001^b^
Length (cm)	44.50 ± 3.14	42.50 ± 3.14	46.75 ± 1.33	0.01^a^
BMI (kg/m2)	14.05 ± 0.61	9.55 ± 0.61	14.09 ± 0.89	0.001^b^
H.C (cm)	36.08 ± 1.88	31.08 ± 1.88	35.08 ± 1.13	0.01^a^
Apgar at 1 min	2.8 ± 0.3	4.8 ± 0.5	5.2 ± 0.1	NS
Apgar at 5 min	5.6 ± 0.6	8.3 ± 0.4	9.1 ± 0.6	NS
Adiponectine (μg/ml)	23.36 ± 5.86	28.2 ± 6.98	36.35 ± 10.55	0.001^b^
Leptin (ng/ml)	22.91 ± 3.73	4.48 ± 3.22	17.02 ± 3.01	0.001^b^
Insulin (μIU/ml)	67.13 ± 8.77	50.92 ± 9.10	55.0 ± 9.19	0.01^a^
Glucose (mmol/L)	4.64 ± 0.65	4.01 ± 0.86	4.21 ± 0.07	NS

Data is presented as n (%) for frequencies and mean ± s.d. for normal continuous variables. ^a^Significant, ^b^highly significant, *NS* not significant. *HIE* Hypoxic Ischemic Encephalopathy, *AGA* Appropriate for gestational age, *SGA* small for gestational age, *M/f* male/female, *NFD/CS* normal vaginal delivery/cesarean section

**Table 2:** Circulating levels of adiponectin, leptin, insulin and glucose in HIE and Non-HIE at birth, 2nd, 6th days

Item		At birth	Day 2	Day 6	p-value
Adiponectine (μg/ml)	HIE	23.36 ± 5.86	23.27 ± 4.93	23.03 ± 5.7	*P* < 0.01^a^
	Non HIE	32.64 ± 9.91	29.22 ± 6.78	26.4 ± 4.98	
Leptin (ng/ml)	HIE	22.91 ± 3.73	22.72 ± 4.73	22.47 ± 3.13	*P* < 0.001^b^
	Non HIE	11.32 ± 7.01	9.98 ± 8.11	9.13 ± 7.22	
Insulin (μIU/ml)	HIE	67.13 ± 8.77	67.33 ± 8.77	67.49 ± 8.77	*P* < 0.01^a^
	Non HIE	53.14 ± 9.30	54.06 ± 4.02	56.18 ± 4.33	

^a^Significant, ^b^highly significant, *NS* not significant. *HIE* Hypoxic Ischemic Encephalopathy, *Non-HIE* Non-Hypoxic Ischemic Encephalopathy

Insulin levels in neonates increased over the study period; they were significantly lower at birth compared to the time point’s 2nd and sixth days of life. SGA circulating insulin level at birth was the lowest while HIE circulating insulin level was the highest in comparison to AGA circulating levels. At birth, 2nd and 6th days, insulin concentrations were comparable between all groups (Table 2).

Intra–group variance revealed that serum levels of adiponectin, leptin and insulin were significantly changed during the first week of life with adiponectin level was the most affected one, while between group variance revealed that neonates with HIE were the most affected group followed by AGA group and lastly SGA as regards these hormonal changes (Tables 3 & 4).

**Table 3:** Circulating levels of adiponectin, leptin, insulin and glucose in HIE and AGA at birth, 2nd and 6th days

Item	Birth		2nd day		6th day	
	HIE	AGA	HIE	AGA	HIE	AGA
Adiponectine (μg/ml)	23.36 ± 5.86	36.35 ± 10.55	23.27 ± 4.93	31.33 ± 9.98	23.03 ± 5.77	28.56 ± 9.38
	*P* < 0.00^b^		*P* < 0.00^b^		*P* < 0.03^a^	
Leptin (ng/ml)	22.91 ± 3.73	17.02 ± 3.01	22.72 ± 4.73	15.98 ± 3.01	22.47 ± 3.13	14.91 ± 2.90
	*P* < 0.00^b^		*P* < 0.00^b^		*P* < 0.00^b^	
Insulin (μIU/ml)	67.13 ± 8.77	55.0 ± 9.19	67.33 ± 8.77	56.98 ± 3.01	67.49 ± 8.77	60.4 ± 11.0
	*P* < 0.00^b^		*P* < 0.00^b^		*P* < 0.00^b^	
Glucose (mmol/L)	4.64 ± 0.65	4.21 ± 0.07	4.33 ± 0.06	4.53 ± 0.38	4.61 ± 0.11	4.36 ± 0.45
	NS		NS		NS	

^a^Significant, ^b^highly significant, *NS* not significant. *HIE* Hypoxic Ischemic Encephalopathy, *AGA* Appropriate for gestational age

**Table 4:** Linear regression analysis of the studied hormones in different studied groups during the first week of life

Item		Confidence interval for B		Standardized Coefficients	P value
		Lower Bound	upper Bound	B	
Non HIE AGA neonate	Adiponectin	0.1	0.015	0.274	0.009^b^
	Leptin	0.33	0.045	2.6	0.011^a^
	Insulin	0.002	0.09	0.214	0.04^a^
Non HIE SGA neonate	Adiponectin	0.147	0.005	0.213	0.07
	Leptin	0.27	0.06	0.15	0.21
	Insulin	0.05	0.06	0.016	0.9
HIE neonates	Adiponectin	0.2	0.022	0.374	0.001^b^
	Leptin	0.13	0.025	1.6	0.01^a^
	Insulin	0.02	0.09	0.224	0.03^a^

^a^Significant, ^b^highly significant, *NS* not significant. *HIE* Hypoxic Ischemic Encephalopathy, *AGA* Appropriate for gestational age, *SGA* small for gestational age

Linear regression analysis was performed using adiponectin as a dependent variable and all parameters measured as independent variables and the body weight and BMI followed by leptin levels were the most predictor variables for adiponectin (*R* = −0.47; *P* = 0.03, *R* = −0.38; *P* = 0.04 and *R* = −0.27; *P* = 0.1 4 respectively) (Not shown in the results).

In all studied groups, the serum adiponectin level was positively correlated at birth with body weight (*r* = 0.509, *p* < 0.001), length (*r* = 0.424, *p* < 0.01), BMI (*r* = 0.460, *p* < 0.001) and leptin levels (*r* = 0.355, *p* < 0.04) but it was not correlated with the insulin level (*r* = 0.160, *p* = 0.277). In neonates with HIE, the serum adiponectin level was not correlated with body weight (*r* = −0.056, *p* = 0.816), BMI (*r* = −0.271, *p* = 0.247), leptin level (*r* = 0.122, *p* = 0.609), or insulin level (*r* = 0.444, *p* < 0.06).) (Table 5).

**Table 5:** Correlations between adiponectin and leptin levels and other studied parameters

Item	HIE		Non-HIE AGA		Non-HIE SGA	
	Adiponectin	Leptin	Adiponectin	Leptin	Adiponectin	Leptin
Gestational age (weeks)	*r* = −0.15	*r* = 0.42	*r* = 0.70	*r* = 0.64	*r* = 0.40	*r* = 0.47
	*p* = 0.71	*p* = 0.32	*p* < 0.001^b^	*p* < 0.01^a^	*p* < 0.01^a^	*p* < 0.01^a^
Weight (Kg)	*r* = −0.05	*r* = 0.36	*r* = 0.60	*r* = 0.93	*r* = 0.50	*r* = 0.73
	*p* = 0.81	*p* = 0.11	*p* < 0.01^a^	*p* < 0.001^b^	*p* < 0.01^a^	*p* < 0.01^a^
Length (cm)	*r* = −0.23	*r* = 0.31	*r* = 0.52	*r* = 0.81	*r* = 0.42	*r* = 0.61
	*p* = 0.96	*p* = 0.18	*p* < 0.01^a^	*p* < 0.01^a^	*p* < 0.01^b^	*p* < 0.01^a^
BMI (kg/m2)	*r* = −0.27	*r* = 0.22	*r* = 0.66	*r* = 0.91	*r* = 0.46	*r* = 0.71
	*p* = 0.24	*p* = 0.350	*p* < 0.01^a^	*p* < 0.001^b^	*p* < 0.001^b^	*p* < 0.01^a^
Insulin (μIU/ml)	*r* = 0.44	*r* = 0.31	*r* = 0.26	*r* = 0.30	*r* = 0.16	*r* = 0.10
	*p* = 0.06	*p* = 0.17	*p* = 0.27	*p* = 0.47	*p* = 0.27	*p* = 0.47
Adiponectine (μg/ml)	----------------	*r* = 0.12	--------------	*r* = 0.55	--------------	*r* = 045
		*p* = 0.60		*p* < 0.0^a^		*p* < 0.02^a^
Leptin (ng/ml)	*r* = 0.12	----------	*r* = 0.55	--------------	*r* = 0.35	--------------
	*p* = 0.60		*p* < 0.04^a^		*p* < 0.04^a^	

^a^Significant, ^b^highly significant, *NS* not significant. *HIE* Hypoxic Ischemic Encephalopathy, *AGA* Appropriate for gestational age, *SGA* small for gestational age

Serum leptin level was positively correlated at birth with body weight (*r* = 0.935, *p* < 0.001), height (*r* = 0.818, *p* < 0.001) and BMI (*r* = 0.918, *p* < 0.001) but it was not correlated with the insulin level (*r* = 0.105, *p* = 0.479) in all studied groups (Table 5).

## Discussion

Analysis of adipose tissue histology of fat cells in newborns demonstrated two populations of cells in the adipose tissue: small cells that do not contain fat and larger cells that contain fat but are small in their diameter compared with adult fat cells [23]. Adiponectin seems to be produced and secreted exclusively by adipocytes and an increase in fat mass leads to down-regulation of adiponectin in adults [24].

Sivan et al. 2003 [25] suggested that neonatal adiponectin is derived mainly from fetal tissues and not from maternal or placental tissues. Therefore, the possibility that a facilitated transport of adiponectin from the maternal blood through the placenta is responsible for the high levels of the hormone in cord blood seems unlikely.

In this study serum adiponectin concentration was significantly lower in neonates with HIE than normal healthy neonates reflecting the injurious effect of hypoxia on the functions of the adipose tissues like other tissues as myocardium, liver, renal and central nervous system [17, 18]. Pardo et al. 2004 [26] speculated that some endocrine, paracrine, and autocrine factor(s) that are responsible for the known suppression of adiponectin production could be reduced in newborns in comparison to older children. Kamoda et al. 2004 [27] found that the levels of serum adiponectin were significantly higher in all newborn neonates than in healthy children.

In this study, the serum adiponectin level was positively correlated at birth with body weight, length and BMI but not with insulin levels and these results are in agreement with many others [28, 29]. This may be due to a lack of negative feedback on adiponectin production resulting from a lack of adipocyte hypertrophy and a low percentage of body fat [30].

In this study, the mean value of serum adiponectin was significantly lower in SGA than in AGA neonates and this may be due to presence of a lower amount of a brown adipose tissue in SGA neonates [27].

In neonates with HIE, the serum adiponectin level was not correlated with body weight, BMI, leptin or insulin levels and this may be attributed to the disturbed mechanisms controlling the growth factors release either from the adipose tissues or from other organs [17, 18].

Leptin is synthesized and secreted in the placenta by the mother and by the fetus [22]. It is detectable from the second trimester and its level increases from the middle of the third trimester towards term, according to the stores of fetal adipose tissue [31, 32].

In this study, leptin and insulin concentrations were significantly higher in neonates with HIE than normal healthy neonates denoting the leptin and insulin insensitivity in these neonates which may be due to the effect of hypoxia and/or ischemia on the peripheral tissues resistance. These results are in consistence with Tzschoppe et al. 2011 [31].

Leptin acts as an endogenous mediator of neuroprotection during cerebral ischemia and exogenous leptin administration protects against ischemic neuronal injury in vitro and in vivo [33, 34]. Leptin and IL-1beta treatment improves neuronal density and decreases apoptosis in the newborn rat and experimental animals with hypoxic-ischemic brain injury [35, 36].

The serum leptin level was positively correlated at birth with body weight, height and BMI but not with insulin level in all studied groups and these results are in accordance with many other studies [37, 38]. In this study we found leptin levels to be higher in AGA neonates with respect to SGA neonates at birth and this is in accordance with others [28, 29]. The previous results were anticipated since leptin is mainly synthesized by the white adipose tissue and released in the circulation proportionally to the amount of body fat mass [39, 40].

Ng et al. 2000 [41] suggested that the low leptin levels in extremely preterm neonates may be a physiologic advantage as body energy utilization can be minimized and nutritional reserves conserved for subsequent growth and development.

Our results revealed that in neonates with HIE, the serum leptin levels were not correlated with body weight, BMI or insulin level after birth denoting the disturbed mechanisms essential for metabolic control.

In this study, serum insulin level was higher in neonates with HIE than normal healthy neonates and it was not correlated with serum adiponectin level and these results are in agreement with others [42, 43].

Iniguez et al. 2004 [43] found no correlations between adiponectin and insulin levels at two years of life, However, Mami et al. 2008 [42] reported negative correlation between adiponectin and insulin at birth and Yamamoto et al. 2002 [44] reported negative correlations between adiponectin and insulin in adults and that serum adiponectin concentration predicts subsequent changes in insulin resistance. This difference with our results may be due to the small sample size, age group of studied patients or the methodology of the study.

In this study, there was no significant difference between SGA and AGA neonates regarding serum insulin levels at birth. This is in agreement with Martinez-Cordero et al. 2006 [28] and with other studies reported that the cord blood c-peptide levels, a proxy for fetal pancreatic insulin secretion, were correlated positively with the birth weight [45, 46], but not with Yajnik et al. 2002 [47] who reported that in Indian neonates, despite their lower birth weight, have hyperinsulinemia.

In this study intra–group variance revealed that serum levels of adiponectin, leptin and insulin were significantly changed during the first week of life with adiponectin level was the most affected one, while between group variance revealed that neonates with HIE were the most affected group followed by AGA group and lastly SGA as regards these hormonal changes.

As expected, in this study all neonatal anthropometric measurements were higher in neonates of AGA than SGA group.

Measurement of the maternal levels of these hormones and correlations between them and neonatal serum levels as well as follow-up of these levels for longer periods (for example for 1 or 2 years) were limitation for this work which need more financial support and large number of patients.

These findings have further clinical implications for pediatricians to understand the effect of hypoxia and or ischemia on different organs of the body including adipose tissue and the different mechanisms controlling its functions.

## Conclusions

Neonates with HIE had lower serum levels of adiponectin and higher serum levels of leptin and insulin than normal healthy neonates at birth and during the early post natal period. SGA neonates had lower serum levels of adiponectin and leptin but not insulin levels than AGA neonates. Positive significant correlations between adiponectin, leptin, body weight and body mass indices were present in normal healthy neonates but not in neonates with HIE reflecting the effect of hypoxia on the regulatory mechanisms controlling the adipose tissue functions.
